# Off-label Augmentation with Cariprazine in a Patient with Schizotypal Disorder and Persistent Suicidal Rumination: A Case Report

**DOI:** 10.1192/j.eurpsy.2026.12151

**Published:** 2026-07-31

**Authors:** S. Mitrovska, S. Arsova, S. Kochoska, A. Risteski, A. Bogdanovska Toskikj, J. Ivanoski

**Affiliations:** 1 Day Hospital; 2 University Clinic of Psychiatry, Skopje, North Macedonia

## Abstract

**Introduction:**

Cariprazine, a D3/D2 and 5-HT1A partial agonist, is approved for schizophrenia, bipolar I disorder, and adjunctive MDD treatment. It’s demonstrated efficacy across positive, negative, and transdiagnostic symptom domains, especially as an augmentative strategy for resistant negative symptoms (Barabassy et al. Pharmaceuticals 2025; 18:995). However its use in schizotypal disorder remains underexplored.

**Objectives:**

To assess the efficacy of cariprazine augmentation to clozapine in a schizotypal disorder case, with focus on negative symptoms, suicidal rumination, and functional outcomes.

**Methods:**

This case report was developed following an unsystematic literature review conducted via databases as PubMed and PsycINFO using keywords including: *cariprazine, clozapine augmentation, negative symptoms, schizotypal disorder*, and *suicidal rumination.* Clinical data were derived from a detailed patient evaluation and follow-up at the Day Hospital, University Clinic of Psychiatry in Skopje.

**Results:**

A 40-year-old woman with a long-standing polymorphic psychiatric illness—marked by affective instability, poor interpersonal functioning, social withdrawal, and multiple suicide attempts and hospitalizations—was admitted for intensification of obsessive suicidal ideation despite high-dose clozapine (up to 400 mg/day) and previous adjunctive SSRIs and risperidone (discontinued due to hyperprolactinemia). On admission to the Day Hospital, clozapine (200 mg/day) was continued, and cariprazine 3 mg/day was initiated, while the patient was included in individual and group psychosocial treatment. Baseline assessment revealed: Positive and Negative Syndrome Scale (PANSS) – Positive 11, Negative 31, General Psychopathology 52; Suicidal Ideation Attributes Scale (SIDAS) 30 (high suicide risk); Personal and Social Performance (PSP) 25/100 (severe dysfunction).After 4 weeks, significant improvement was noted in regards to: PANSS – Positive (−18%), Negative (−42%), General (−27%) ; SIDAS 21 (−30%) reduction; PSP 65/100, indicating moderate recovery. Clinically, obsessive suicidal ruminations subsided, affective responsiveness improved, and the patient resumed self-care, parenting roles, and active participation in individual, group, and family therapy.

**Conclusions:**

The rapid reduction in negative symptoms and obsessive suicidal ideation following cariprazine augmentation supports its broad efficacy, likely mediated by high D3 receptor affinity and serotonergic activity. While clozapine remains the gold standard for treatment-resistant psychosis, a substantial proportion of patients respond only partially. This case contributes to the limited evidence suggesting that cariprazine may enhance clinical and functional outcomes in schizotypal disorder, particularly where suicidality and negative symptoms predominate. Further research is needed to clarify its therapeutic role in this population.

**Disclosure of Interest:**

None Declared

